# Evaluating the current state of the art of Huntington disease research: a scientometric analysis

**DOI:** 10.1590/1414-431X20176299

**Published:** 2018-01-11

**Authors:** L.A. Barboza, N.C. Ghisi

**Affiliations:** Laboratório de Biologia Molecular, Universidade Tecnológica Federal do Paraná, Dois Vizinhos, PR, Brasil

**Keywords:** Genetic disease, Chorea, CAG repeat, Neurodegeneration, HD gene

## Abstract

Huntington disease (HD) is an incurable neurodegenerative disorder caused by a dominant mutation on the 4th chromosome. We aim to present a scientometric analysis of the extant scientific undertakings devoted to better understanding HD. Therefore, a quantitative study was performed to examine the current state-of-the-art approaches that foster researchers’ understandings of the current knowledge, research trends, and research gaps regarding this disorder. We performed literature searches of articles that were published up to September 2016 in the “ISI Web of Science™” (http://apps.webofknowledge.com/). The keyword used was “Huntington disease”. Of the initial 14,036 articles that were obtained, 7732 were eligible for inclusion in the study according to their relevance. Data were classified according to language, country of publication, year, and area of concentration. The country leader regarding the number of studies published on HD is the United States, accounting for nearly 30% of all publications, followed by England and Germany, who have published 10 and 7% of all publications, respectively. Regarding the language in which the articles were written, 98% of publications were in English. The first publication to be found on HD was published in 1974. A surge of publications on HD can be seen from 1996 onward. In relation to the various knowledge areas that emerged, most publications were in the fields of neuroscience and neurology, likely because HD is a neurodegenerative disorder. Publications written in areas such as psychiatry, genetics, and molecular biology also predominated.

## Introduction

Huntington disease (HD) is a progressive neurodegenerative disease that belongs to a unique group of autosomal-dominant disorders. This disorder is caused by CAG trinucleotide repeats in the 5′ coding region of the IT15 (Interesting Transcript15) gene located on locus 4p16.3 (1). HD expanded alleles have more than 36 CAG units in the HD gene, whereas normal individuals have from 10–35 CAG units (2). This mutation generates a functionally defective protein called huntingtin (HTT), a protein of uncertain molecular function(s) (3,4). HTT is a ubiquitously expressed protein that is located throughout the body. Mutant HTT, which contains pathologically extended polyglutamines, causes the earliest and most dramatic neuropathological changes in the neostriatum and cerebral cortex (3), whereas a loss of wild-type HTT function contributes to disease development (5–9).

Extended polyglutamines confer structural conformational changes in HTT, which yields novel properties and results in aberrant interactions with multiple cellular components (10,11). The diverse and variable aberrations mediated by mutant HTT perturb many cellular functions that are essential for neuronal homeostasis and that underlie the pleiotropic mechanisms of HD pathogenesis (3,9).

As autosomal dominant inherited diseases, there is theoretically a 50% chance of inheritance for each child of a heterozygote person (12,13). The nature of the mutation, which increases in successive generations, leads to non-Mendelian inheritance patterns due to the presence of unstable and abnormal expansions of DNA triplets (trinucleotides) (14). Unfortunately, there is no definitive cure for HD; patients can only be treated to minimize symptoms, thus promoting a better quality of life (15). The clinical symptoms of HD include involuntary movements (called chorea), progressive dementia, and severe weight loss, all of which inescapably progress to death within one or two decades after the initial symptoms emerge (10,16).

Neuropsychiatric symptoms, including depression, apathy, and irritability, may already be present many years before any motor symptoms appear (17). All of these symptoms can be both erroneously interpreted and inadequately treated. In the interval between symptom onset and treatment, depression is common; this may be dangerous due to the risk of increased suicidal behaviors among those affected. In fact, it has been estimated that patients with HD may present rates of suicidal behaviors four times greater than the general population (18).

The precise timing of the clinical diagnosis of HD is poorly characterized and is primarily based on the presence of motor symptoms (19). For this reason, making a correct diagnosis is highly critical, and it should be achieved with molecular confirmation of the repeat length. According to the Technical Standards and Guidelines for Huntington Disease Testing, allele sizes of ≤26 CAG repeats have never been associated with an HD phenotype. Allele sizes of 27–35 CAG repeats are rare and have not been convincingly associated with an HD phenotype. However, allele sizes of 36–39 CAG repeats have been reported in both clinically affected and clinically unaffected individuals (20). Alleles with more than 40 CAGs present 100% penetrance for HD (21). Due to the gloomy prognosis of HD, all positive results should serve as an indication for genetic counseling, and testing should be available for other at-risk family members. In relation to molecular diagnosis, articles discussing several sets of primers, as well as various polymerase chain reaction (PCR) conditions, methods of separation, and detection techniques were published in the 1990s, and these approaches have been optimized in later years. It has been noted that assay conditions and post-PCR analyses should be optimized to ensure the accurate and unambiguous quantitation of repeat length (22–28).

Thus, the overall aim of this study is to perform a scientometric analysis of the current scientific approaches devoted to HD. Specifically, we provide a quantitative investigation into the current scientific approaches used in HD to develop a greater understanding of the existing knowledge, as well as to determine the main trends and gaps in the research on this disorder.

## Material and Methods

We used datasets from the Thomson Institute for Scientific Information (“ISI Web of Science™”) (http://apps.webofknowledge.com/). A search using the keyword “Huntington disease” retrieved 14,036 articles. The analysis was based on the abstracts of papers published between 1945 and October 2016. These were refined based on relevance, resulting in a final number of 7732 results. The papers were then categorized according to the language in which they were written, as well as by the country, year, and field of study. These data were included in a spreadsheet, thus enabling the analysis by a comparative graph and various tables.

## Results

**Figure 1.**
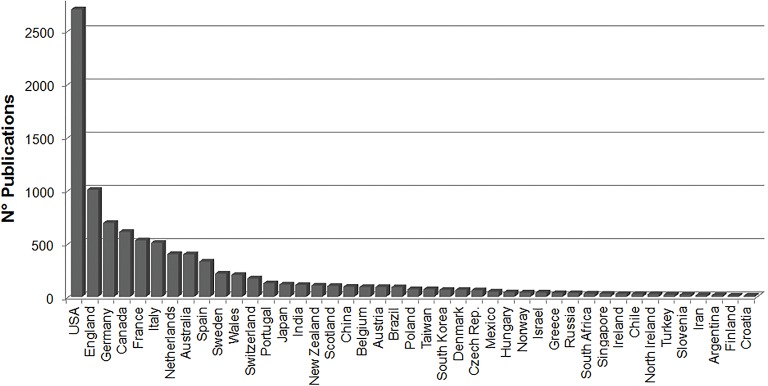
Countries with the highest percentage of publications (up to 10 publications included) on Huntington disease.

Through this analysis, the countries that invest the most resources in and publish more articles about HD were identified. Figure 1 presents countries that published a minimum of 10 publications on HD. There were 92 countries that had published or collaborated on research related to HD. The United States is the world leader in terms of the number of studies published on HD, with 2700 articles, accounting for more than one quarter of the world’s publications on this disorder (28.12%). England ranks second (10%), followed by Germany (7%) and Canada (6%). Emerging countries, such as India, only appear after the 15th position. Among the South-American countries, Brazil is the most prominent, as it holds the 21st position with 89 publications, surpassing European countries such as Poland and Denmark (70 and 64 publications, respectively). Among the Latin-American countries, Mexico follows Brazil in 28th position, with 48 publications.

**Figure 2.**
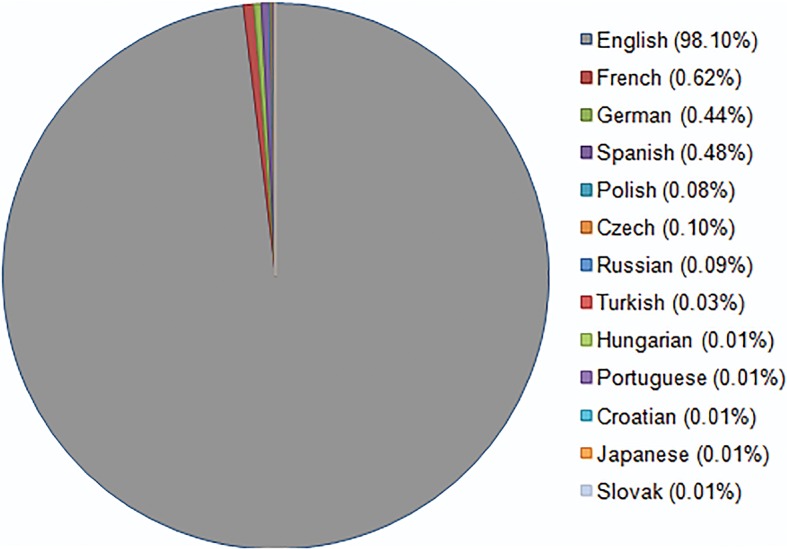
Graph showing the languages that have been used in publications about Huntington disease.

In Figure 2, the graph clearly shows that 98% of the publications are written in English, even those published in countries that have a different official language. Articles written in other languages appear in much smaller proportions, such as French, German, Spanish, Polish, and Czech, all representing fewer than 1% of the total publications on the topic.

**Figure 3.**
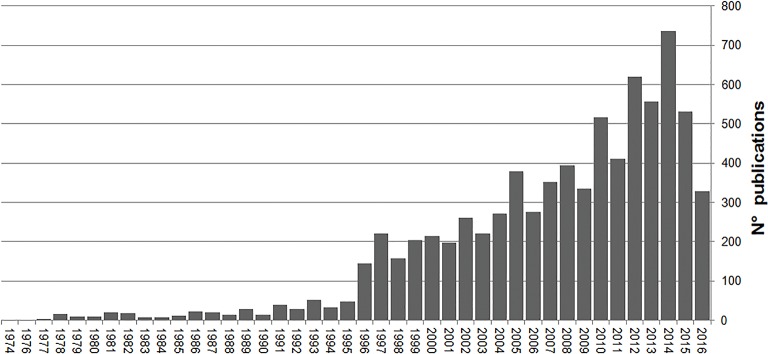
Number of articles on Huntington disease published each year from 1974 to 2016.

The first publication recorded for HD was published in 1974, and there were no records of HD-related works in 1975; from 1976 until the present day, new papers have been published on the subject every year, as demonstrated by an increasing scale (Figure 3). The great surge in the number of articles published since 1996 is noticeable, where a significant number of studies were published on the approaches used for treating this disease. In 1995, 47 papers were published on this subject, whereas in the next year, this value more than tripled to 145 publications. The largest number of publications on HD can be seen in 2014; there was a decrease in the number of publications on HD in 2015, and an even smaller number was published in 2016, likely because the publications for the current year have not yet been finalized.

**Figure 4.**
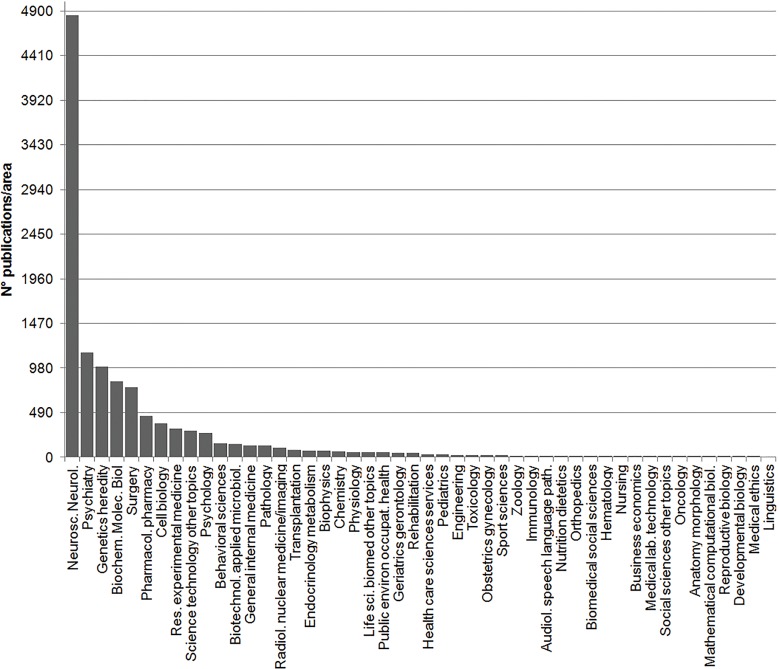
Number of publications classified by areas of knowledge. Areas have been abbreviated according to International Index Medicus.

Ninety-one different fields of study were identified. Almost half of the published articles fell within the field of neuroscience and neurology (41%; Figure 4), while 10% percent of publications were published in psychiatry, followed by 8% in hereditary genetics, biochemistry and molecular biology (7%), and surgery (7%).

## Discussion

Our results showed the trends in publication related to country of origin and the fields of study of HD-related published articles around the world. It is evident that the United States is a strong leader in terms of the number of publications on the subject, likely due to the availability of greater financial and trained human resources available in the country. One important factor that increases the development of various initiatives on HD-related research in specific countries is the prevalence rate of this disorder in a given country. Epidemiological studies of HD report a wide variety (more than ten-fold) in the prevalence rates of the disease around the world (29). In fact, there are between 5 and 10 affected people per 100,000 members of the general population in most Western European countries, as well as in the United States and Canada (29). Canada appears in the fourth position in terms of the number of HD-related publications on HD.

The original HD mutation was considered to have its origins in Western Europe and, subsequently, it was believed to have spread around the world, largely through emigration (12). Consequently, in various populations, including Chinese, Japanese, African Blacks, and Finnish populations where there is a reduced rate of Western European admixture, one might expect the frequency of HD to be lower (30). In Japan, for example, the prevalence rate was determined to be from 0.11–0.45 per 100,000 individuals (30). Consequently, less research about this issue has been developed in these countries. For instance, Japan occupies the 20th position in number of studies published about HD.

It is important to note that the number of publications may be strongly associated with the economic or financial situations of a given country. Countries with emerging economies, such as Latin America, have neither investments nor skilled labor to advance in research as a developed country. In Latin American countries, HD shows a wide range of prevalence rates, with 4 in 100,000 affected in Mexico City (31) and 1 of 200,000 affected in the general population of Venezuela (32). When ranking the number of papers published on HD, these countries appear in 29th and 44th position, respectively. Furthermore, the limited number of papers found on this disease suggests that HD is poorly diagnosed in Latin America (33).

On the other hand, there are two hotspots in South America that feature an extraordinarily high prevalence rate of HD; the Indian-White inhabitants from the Valley of Caãete in Peru, with a prevalence rate of 1 of 143 individuals (34), and on the Western coast of Lake Maracaibo, Zulia State, Venezuela, with 1 person affected among every 23,000 people (32,35). These high prevalence rates are probably due to the founder effect, as cited by Adachi and Nakashima (36). The founder effect is a common outcome when new populations are established from a small number of founding individuals (37). These founding individuals carry with them only a fraction of the genetic diversity of their parents; therefore, the founder effect results in decreased genetic diversity and distinctive allele frequency patterns in the newly established population (37). The founder effect can thus increase the frequency with which certain rare disorders occur, as exemplified by HD in this regions of Venezuela and Peru. In Lake Maracaibo in Venezuela, the common ancestor of subsequently affected families was likely a woman who had 10 children about 200 years ago. During this time, the locals named Huntington disease “*el mal de San Vito*” (Saint Vitus’s disease) (38).

When ranking the number of published HD studies, Brazil is in first place among South American countries. Given the size of Brazil, there are no definitive figures regarding the prevalence of HD across the entire country. However, there are specific regions where HD is most prevalent, such as in Feira Grande in Northern Brazil. There, researchers identified 22 cases of HD within the total population of 22,000. This means that in this region, 1 in 1000 individuals will develop HD. The authors attribute this rate to the high amount of marriages between cousins in the region (39).

Another town in Brazil called Ervalia, in the State of Minas Gerais, has a high prevalence of HD because, historically, this place was a social refuge for people with the disease and their families, who migrated from more developed neighboring cities to this rural city (40). As an outcome, this disease carriers that immigrated married within the community, without any genetic counselling, leading to the high prevalence of the HD (40). Moreover, many patients from Ervalia were misdiagnosed with Alzheimer’s and Parkinson’s disease, which contributed to the misinformation among the risk group further increasing the number of cases in the city (40).

When examining the languages in which these studies were published, one can notice the overwhelming majority of publications written in the English, which ultimately reinforces that English has been established as the international language of science. We can cite the articles of Hutzinger (1989) and Heinrich (2010) (41,42), titled “Publish - In English - Or Perish”, which highlight the need to internationalize knowledge through a universal language. Only 2% of publications are not written in English; 12 other languages are used, most of which are of European origin, supporting the finding of the higher incidence of HD in these countries.

In our research, the first report of HD found in the Institute for Scientific Information (ISI) was published in 1974. However, this disease was described more than 100 years ago by George Huntington in the paper titled “On Chorea”, published in 1872 (43); only later was this disease referred to as “Huntington Disease”. In his paper, Huntington describes the principal symptom of HD - chorea - as a disease of the nervous system (43). He explains that the name chorea was assigned due to the tendency of affected patients to dance - that is, these patients exhibited involuntary movements (43). Huntington already reported on the hereditary nature of chorea, as well as on two other marked peculiarities of this disease: the development of mental illness and suicidal behaviors, as well as its eventual progression to death during adulthood (43). Despite the fact that he was unable to explain the correct genetic mechanisms underlying this disease, particularly since Mendel’s study (published in 1865) was practically unknown until 1909, some of the characteristics he did note were remarkable: the disease never skips generations; once a branch of the family does not present the disease, it will not reappear in that branch (43).

Upon searching historical databases, we found that before the 1900s, other studies reported cases of Huntington’s chorea. According to Ramírez et al. (44), in 1890, Aróstegui described cases of HD in Cuba; in 1891, Couto cited cases in Brazil, while Costa discussed cases in Argentina in 1894. Only as recently as 1983 was the HD gene mapped in chromosome 4, locus 4p16.3; this was a novel finding, as it was the first gene to be mapped onto a human chromosome without any prior indication of the gene’s location (45). Finally, HD was cloned in 1993, and thus its pathogenic mutation could be identified: it involved an expansion of CAG repeats in the first exon of the *HTT* gene. In normal individuals, the number of CAG repeats averages between 17 and 20 units. Alleles with <26 CAG repeats are normal, whereas those with between 27 and 35 repeats are classified as intermediate or meiotically unstable alleles (21,46). Alleles with 36–39 CAG repeats have a reduced penetrance and often develop HD with a later onset, while those with >40 CAGs are at risk for HD with 100% penetrance (21). Approximately 99% of alleles show heterozygosis (21), and some authors agree that there is an irregular inverse correlation between repeat length and the age of HD onset (47,48). According to the study of Ruocco et al. (49) conducted with Brazilian patients, there was a significant correlation between age at onset of HD and the length of the CAG repeat; there was also a relationship between clinical disability and age at onset.

HD is a neurodegenerative disease, and, for this reason, we can see the concern about the issue in specific fields of publications. Most HD-related studies (41%) are published in the neuroscience and neurology field. This reflects the associated concerns about the symptoms of this disease. HD pathology involves a loss of medium spiny neurons in the striatum, and the progressive neurodegeneration associated with this disease affects the striatum and other brain regions (50). A stereological comparison of the striatal neurons of healthy individuals and HD patients has highlighted that there is an enormous decrease of ∼88% of neurons in the striatum of HD patients (51). Moreover, the degree of basal ganglia atrophy was correlated with the length of the CAG repeat. However, there were no correlations between cerebellar or cerebral volume and the length of the CAG repeat (49).

The second most frequent field related to HD is psychiatry. As reported by Huntington, HD, as a movement disorder, is accompanied by numerous personality changes (nervous excitement) and cognitive decline (a tendency toward mental illness). Huntington also observed that there was an increased incidence of suicide rates among patients (43). Furthermore, many HD-related papers are also published in the genetic and molecular biology field given the need to better understand the molecular mechanisms of disease development. Since HD affects multiple cellular processes, the molecular mechanisms of pathogenesis should be investigated at multiple levels.

The HD gene encodes a large, 3,144-amino-acid-long protein, the previously explained huntingtin (HTT), which plays numerous roles in a variety of cellular functions, the most prominent of which are vesicle trafficking, energy production, and transcription (8,21). The CAG repeat in the HD gene encodes an expansion of glutamine residues in the N-terminus of HTT, beginning after the 17th amino acid (21). The mechanism by which the expressed expanded CAG repeat in HTT causes HD is still poorly understood. However, it is known that mutant HTT is prone to aggregation in neurons (52). The expression of long glutamine tracts - either alone, in the context of an N-terminal fragment or full-length HTT, or inserted into other proteins - has been shown to disrupt a wide variety of biological functions within cells (21).

Recent studies indicate that autophagy is related to various pathological conditions, including neurodegenerative diseases such as HD and Parkinson’s disease (53). Autophagy is an important biological process that is essential for the removal of injured organelles and toxic or aggregated proteins, as they are delivered to the lysosome for degradation (54). Long-term studies, such as those developed by the 2016 Nobel Prize winner, Yoshinori Ohsumi, have identified the genes involved in the autophagy pathway, and they can help us to better understand this process in both normal physiology and neurodegenerative diseases (55). HD is a unique neurodegenerative proteinopathy, insofar as autophagy is dysfunctional, and wild-type HTT also appears to play several roles in regulating the dynamics of autophagy (54). It has been shown that, autophagy is affected at several steps in HD, as there is a defect in cargo loading, in the trafficking of autophagosomes, and in the decreased fusion between autophagosomes and lysosomes, leading to a build-up of toxic materials in the cytoplasm and empty autophagosomes (54). Thus, autophagy has become potentially a primary target for the treatment of neurodegenerative diseases, including HD, which involve protein aggregation (54).

Despite all efforts, HD is still an incurable brain disorder. Historically, HD has been an easy disorder to define; however, it is surprisingly difficult to find effective cures (56). Furthermore, there is no therapy for slowing down degeneration or reducing the rate of clinical decline. Currently, this seems to represent the majority of the existing HD research. Some symptoms can be treated pharmacologically, while others can only be addressed via supportive non-pharmacological measures (57). Conversely, novel therapeutic strategies are in development, such as RNA interference (RNAi), antisense oligonucleotide, ribozyme, DNA enzyme, and genome-editing approaches, while engineered antibody therapies that aim to silence or repair the mutant HTT gene hold great promise (38,58–61). Furthermore, several studies examining the use of transplantation in animal models have been published. Transplantation appears to be an emergent knowledge area, with approximately 30 articles published on this topic. Most of these studies are still in the experimental phase and they are currently being tested *in vitro* or in animal models. In animals, striatal precursors, which are differentiated *in vitro*, are transplanted to the striatum; this approach has reportedly improved disease phenotypes (50).

In conclusion, the research on HD has increased remarkably over the years. Several methods and therapies are currently in development to address the etiology, symptomatology, and progression of this disease. However, at present, HD patients and their families must wait and hope for rapid advances in science and medicine to eventually minimize the level of suffering among those affected.
